# Facile Fabrication of Highly Hydrophobic Onion-like Candle Soot-Coated Mesh for Durable Oil/Water Separation

**DOI:** 10.3390/nano12050761

**Published:** 2022-02-24

**Authors:** Jiajia Song, Na Liu, Jiakai Li, Yingze Cao, Haijie Cao

**Affiliations:** 1 Institute of Materials for Energy and Environment, College of Materials Science and Engineering, Qingdao University, Qingdao 266071, China; songjiajia540819@163.com (J.S.); 2019025733@qdu.edu.cn (J.L.); 2 Department of Chemistry, Tsinghua University, Beijing 100084, China; liun12@tsinghua.org.cn; 3 Qian Xuesen Laboratory of Space Technology, China Academy of Space Technology, Beijing 100094, China

**Keywords:** highly hydrophobic, onion-like candle soot, filtrating material, oil/water separation, reusability and durability

## Abstract

Although sundry superhydrophobic filtrating materials have been extensively exploited for remediating water pollution arising from frequent oil spills and oily wastewater emission, the expensive reagents, rigorous reaction conditions, and poor durability severely restrict their water purification performance in practical applications. Herein, we present a facile and cost-effective method to fabricate highly hydrophobic onion-like candle soot (CS)-coated mesh for versatile oil/water separation with excellent reusability and durability. Benefiting from a superglue acting as a binder, the sub-micron CS coating composed of interconnected and intrinsic hydrophobic carbon nanoparticles stably anchors on the surface of porous substrates, which enables the mesh to be highly hydrophobic (146.8 ± 0.5°)/superoleophilic and resist the harsh environmental conditions, including acid, alkali, and salt solutions, and even ultrasonic wear. The as-prepared mesh can efficiently separate light or heavy oil/water mixtures with high separation efficiency (>99.95%), among which all the water content in filtrates is below 75 ppm. Besides, such mesh retains excellent separation performance and high hydrophobicity even after 20 cyclic tests, demonstrating its superior reusability and durability. Overall, this work not only makes the CS-coated mesh promising for durable oil/water separation, but also develops an eco-friendly approach to construct robust superhydrophobic surfaces.

## 1. Introduction

Ever-growing water pollution, caused by frequent oil spills generated from petroleum exploration and oily wastewater emission in daily life activities, has severely threatened the human health and aroused worldwide environmental concerns [1,2]. Hence, it is of great significance to develop high-performance oil/water separation materials to achieve the rapid and efficient removal or collection of oils from water [3]. Generally, the produced oily wastewater can be classified into two types: immiscible mixture and stable emulsion [4,5]. Interconnected porous materials are promisingly utilized for tackling oil/water mixtures, ascribed to their large porosity, low cost, and flexibility of operation [6,7,8,9]. In addition, potential separation materials should have the opposite wettability to water and oil [10,11].

Inspired by lotus leaves with excellent water-repellent and self-cleaning properties, the biomimicry of superhydrophobic surfaces has drawn much attention in academic and industrial fields for applications in self-cleaning [12,13], oil/water separation [14,15], directional water transport [16], anti-icing [17], anticorrosion [18], and drag reduction [19,20]. Principally, hierarchical micro/nanostructures and inherently low surface energy chemicals are the two characteristics needed to construct superhydrophobic surfaces [21,22]. Superhydrophobic materials for the separation of oil and water can usually be divided into superhydrophobic absorbing materials and superhydrophobic filtrating materials [23]. Compared with the superhydrophobic absorbing materials which still require further squeezing operation [24,25,26,27], superhydrophobic filtrating materials have gained more popularity because of the simpler operation, high separation efficiency, and penetration flux [28,29,30,31,32,33]. Superhydrophobic filtrating materials are typically fabricated via a two-step process: creating a hierarchical roughness on the surface of a porous substrate and then physically or chemically modifying the substrate with a low surface energy material [34]. To date, considerable methods have been put forward to develop the superhydrophobic filtrating materials. However, most of them need rigorous reaction conditions, expensive reagents, and bring about the formation of unstable layers between the substrate and hydrophobic molecules, which is undesirable in scale-up industrial production.

Arising from the attractive morphology, high specific surface area, low toxicity, good biocompatibility, and inherent chemical inertness [35], carbonaceous nanomaterials possess novel physical, chemical, and mechanical properties, and can be harnessed for wide applications, such as sorbents [36], optoelectronics [37], environmental sensors [38], and cell imaging [39]. As a bottom-up synthesis route, candle soot (CS) can be readily obtained from incomplete combustion of a paraffin candle flame, which is non-cumbersome and cost-effective [40]. As a carbon material with promising morphology, CS is conducive to the fabrication of superhydrophobic surfaces. Besides, making this common waste profitable rather than threatening the environment and human health is desirable and in accordance with the goal of achieving carbon neutrality. Nevertheless, further doping or modification with low surface energy materials is a requisite for possessing robust super-hydrophobicity due to the weak inter-particle interactions and poor adhesion of the CS layer with substrates [41,42]. To this end, Li et al. prepared a superhydrophobic sponge by dip-coating the CS and hydrophobic SiO_2_ nanoparticles on the sponge skeletons, where polyurethane resin was added to enhance the mechanical strength [43]. Khosravi et al. synthesized a superhydrophobic and superoleophilic steel mesh by deposition of CS nanospheres on the mesh, followed by vapor phase deposition of polypyrrole [44]. Although these enlightening advancements have been made in terms of improving the robustness of CS-based superhydrophobic materials to achieve oil/water mixtures’ separation or the removal of oils and organic solvents from water, there is still considerable interest in exploiting more simplified and inexpensive fabrication strategies.

Herein, we present a simple and cost-effective method to fabricate the highly hydrophobic mesh based on CS nanoparticles. Taking advantage of a superglue acting as a binder, the CS-coated layer is ultrathin and stable. The as-prepared mesh exhibits high hydrophobicity and superoleophilicity, as well as resistance to harsh conditions, enabling efficient and durable separation of light or heavy oil/water mixtures with high separation efficiency and low water content. In addition, the CS-coated mesh maintains excellent separation performance and stable hydrophobicity after 20 repeated separation tests, exhibiting superior reusability and durability. More importantly, such strategy can be promisingly applied to develop robust superhydrophobic surfaces in practical applications.

## 2. Materials and Methods

### 2.1. Materials

Stainless-steel mesh (SSM, 400 mesh) was supplied from Anping Stainless-Steel Mesh Manufacturer, Hebei, China. Commercial paraffin candles were purchased from the local market (Qingdao, China) EVO-STIK Serious Glue was purchased from Bostik Co., Ltd., Shanghai, China. Diesel was obtained as a commercial product and used as-received. Hexane, petroleum ether (PE), toluene, chloroform (CHCl_3_), and carbon tetrachloride (CCl_4_) were of analytical grade from Sinopharm Chemical Reagent Co., Ltd., Beijing, China.

### 2.2. Fabrication of Glued SSM

The SSM was first ultrasonically washed by immersing in deionized water and acetone, respectively, for 15 min to remove contaminants. Then, 0.5 g of EVO-STIK Serious Glue was added into 50 mL of ethanol and vigorously stirred for 30 min to form a translucent solution. The cleaned SSM was dipped in the glue solution for 10 min under ambient conditions and then dried at 80 °C to obtain a glued SSM.

### 2.3. Fabrication of CS-Coated Mesh

**Figure 1:**
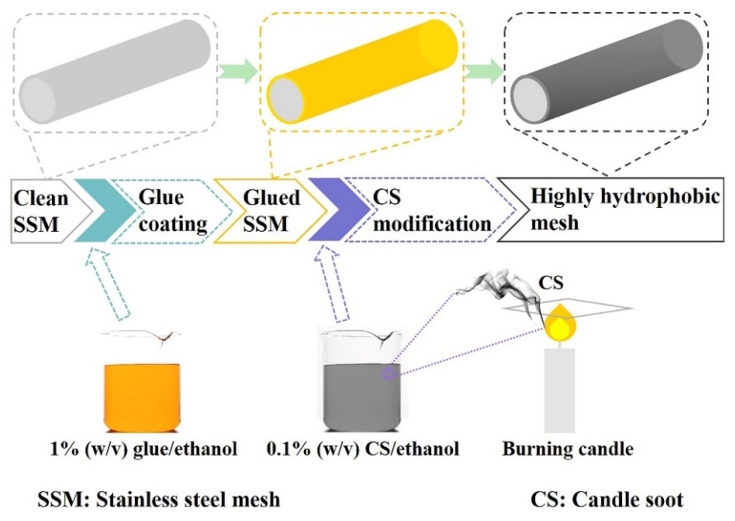
Preparation process of the highly hydrophobic CS-coated mesh.

Candle soot (CS) was synthesized by incomplete combustion of a paraffin candle flame. Briefly, a commercial candle was lit in air, and a silicon wafer was placed above the wick to collect the soot produced, which was used directly without further processing. Highly stable homogeneous CS dispersion was prepared by simply mixing 50 mg of CS in 50 mL of ethanol followed by sonication for 15 min. Subsequently, the pre-obtained glued SSM was immersed into the CS dispersion and the modification reaction was carried out at room temperature under 200 rpm for 16 h, as illustrated in Figure 1. For comparison, the CS-coated meshes were also fabricated according to the same procedure by reacting for 5 min and 1 h, respectively. The final meshes were obtained after being dried at 80 °C for 1 h.

### 2.4. Characterization

The surface morphologies of the meshes and particle size of the CS were observed using the field emission scanning electron microscope (FESEM, JEOL JSM-7800F, Tokyo, Japan) and transmission electron microscopy (TEM, JEOL JEM-2100, Tokyo, Japan). The elemental analysis and chemical composition of the glued SSM and CS-coated mesh were obtained by energy dispersive spectroscopy (EDS) and X-ray photoelectron spectroscopy (XPS, PHI5000 Versaprobe III, Kanagawa, Japan). X-ray diffraction (Rigaku Ultima IV, Tokyo, Japan) was used to record the microstructures of the glued SSM and CS-coated mesh. Raman spectra of the glued SSM and CS-coated mesh were obtained using Raman spectroscopy (Renishaw inVia Raman spectrometer, New Mills, UK). The Brunauer–Emmett–Teller (BET) specific surface area and the pore size distribution of CS powders were determined from the nitrogen adsorption/desorption isotherms obtained by Autosorb iQ3 (Quantachrome, Boynton Beach, FL, USA) at 77 K. The surface wettability of the samples was tested with a contact angle measurement instrument (BOEN-6489, Feierboen Industry Development (Shanghai) Ltd. China). The static contact angle of each sample was obtained by measuring at least five contact angles at different positions and calculating the average. Water content in the collected filtrates after each separation was measured by an automatic trace moisture analyzer (KFC-10, Zibo Aiji Electric Co., Ltd., Zibo, China).

### 2.5. Oil/Water Separation Experiment

In this study, various oil/water mixtures were prepared by mixing 20 mL of organic solvents with a density lower or higher than water (hexane, PE, toluene, CHCl_3_, and CCl_4_) and 20 mL of water, and then magnetically stirred for 15 min, respectively. A homemade device was utilized during the oil/water separation test, where the as-fabricated meshes were fixed between two glass tubes. For each separation, the prepared oil/water mixture was poured into the upper glass tube and quickly separated by the mesh. To test the stability of CS-coated SSM, the separation experiments of the hexane/water mixture and the CCl_4_/water mixture were repeated for 10 cycles, respectively. After one filtration, the mesh was rinsed with plenty of ethanol and dried for the next cycle. The separation efficiency was calculated according to Equation (1):

H = (1 − C_p_/C_o_) × 100% (1)

where η, C_p_, and C_o_ represent the separation efficiency, water concentration in the collected filtrate, and water concentration in the original mixture, respectively.

## 3. Results and Discussion

### 3.1. Surface Morphology and Chemical Component Characterizations

**Figure 2:**
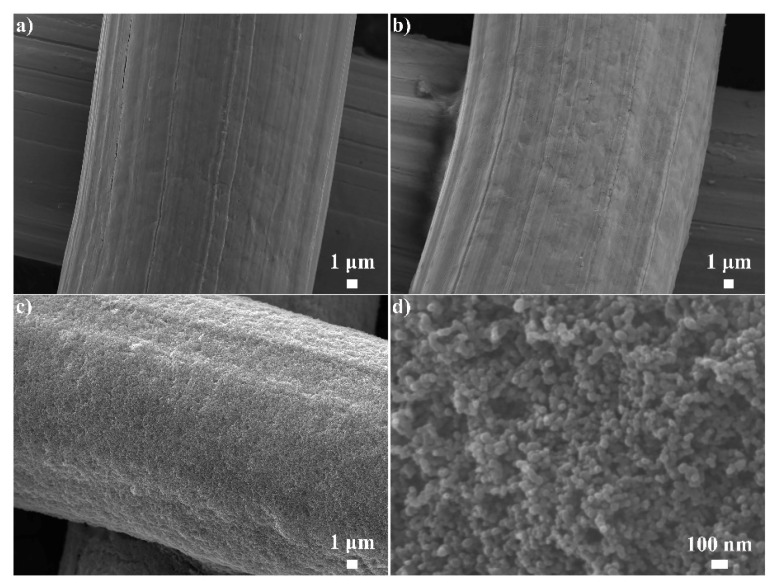
SEM images of (**a**) the pristine SSM, (**b**) glued SSM, and (**c**,**d**) CS-coated mesh with low and high magnifications.

Figure 2a presents the typical morphology of the pristine SSM, which is knitted by stainless-steel wires with an average size of 26.7 μm. The SSM surface was relatively smooth except for some texture distributed on the surface, which was maybe created by abrasion. The glue used in this work is a silane-modified polyether, which has become increasing popular because it is environment-friendly, waterproof, weather resistant, and so on. Additionally, the low surface energy and high penetrability endow the glue with excellent adhesion to various inorganic, metal, and plastic substrates [10]. After moisture curing, a network structure connected by a flexible polyether long chain will be formed with the Si-O-Si bond as a cross-linking point. The long-chain structure can afford a platform for bonding different materials based on the surface energy similarity. As shown in Figure 2b, the stainless-steel wires were covered with a thin glue layer. In Supplementary Figure S1, the SEM mapping images of glued SSM displayed homogeneous distributions of C, Si, and Fe elements, indicating the uniform deposition of glue molecules. After being coated with CS for 16 h, the wires displayed an average diameter of 28.2 μm (Figure 2c), suggesting that CS was successfully coated on the glued SSM, and the thickness of the formed coating was about 750 nm. The high-magnification SEM image (Figure 2d) showed that the entire micrometer-scale wire surface was coated with an irregular nanoparticle network comprised by even particle-size distribution in the micro/nanoscale rough structure required by the superwetting property. Supplementary Figure S2 shows the effect of coating times on the changing morphology of the fabricated meshes at ambient temperature. When the reaction time was 5 min, disordered and sparse micron-to-submicron-scale clusters were observed on the wire surface. When the reaction time increased to 1 h, the coverage of the CS coating significantly increased. Obviously, the coating time of CS is critical for depositing enough CS particles to ensure the surface hydrophobicity and roughness.

**Figure 3:**
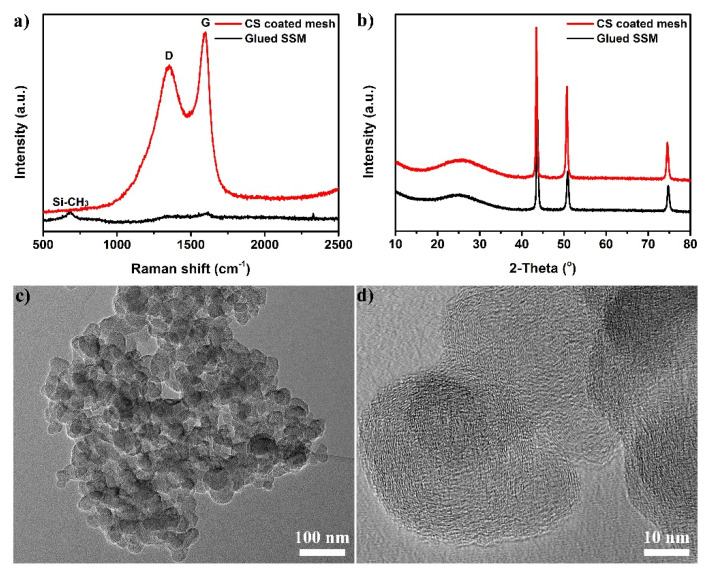
(**a**) Raman spectra and (**b**) XRD patterns of the glued SSM and CS-coated mesh. (**c**,**d**) Low- and high-magnification TEM images of the CS product.

Raman spectroscopy examination was conducted to analyze the molecular structures of the as-prepared mesh, as shown in Figure 3a. The spectrum of glued SSM showed a relatively strong peak at 679 cm^−1^ arising from the Si-CH_3_ symmetric rocking mode of silane-modified polyether. In the Raman spectrum of CS-coated mesh, two broad peaks were observed at ~1353 and 1596 cm^−1^ attributed to graphite’s D and G bands, respectively. The D band of CS was attributed to the presence of amorphous carbon and surface defects, while the G band corresponded to the graphite phonon mode, suggesting that the CS coating is composed of crystalline graphitic carbon. The relative intensity ratio of D and G bands was calculated as 0.82, indicating the highly disordered feature in the as-prepared sample. The XRD pattern of glued SSM in Figure 3b matched well with the cubic phase, with the characteristic peaks at 2θ = 43.5, 50.7, and 74.5° corresponding to the (111), (200), and (220) planes of metallic Fe (JCPDS No. 52-0513). The broad peak at 25.0° with low intensity could be attributed to the diffraction of the amorphous structure of silane. It is clear that no peak for CS was observed, demonstrating that large amounts of amorphous carbon and unburned wax exist in the CS-coated mesh. Figure 3c,d display the low- and high-magnification TEM images of the collected CS, which clearly show that the product is composed of spherical nanoparticles with average diameters ranging from 20 to 55 nm in the form of closely packed agglomerates. The high-resolution TEM image of overlapped nanoparticles revealed that the CS exhibited an interconnected and onion-like graphitic structure. The specific surface area of the collected CS powders was also calculated to be about 63.3 m^2^ g^−1^ according to the N_2_-sorption isotherm measured at 77 K (Supplementary Figure S3).

**Figure 4:**
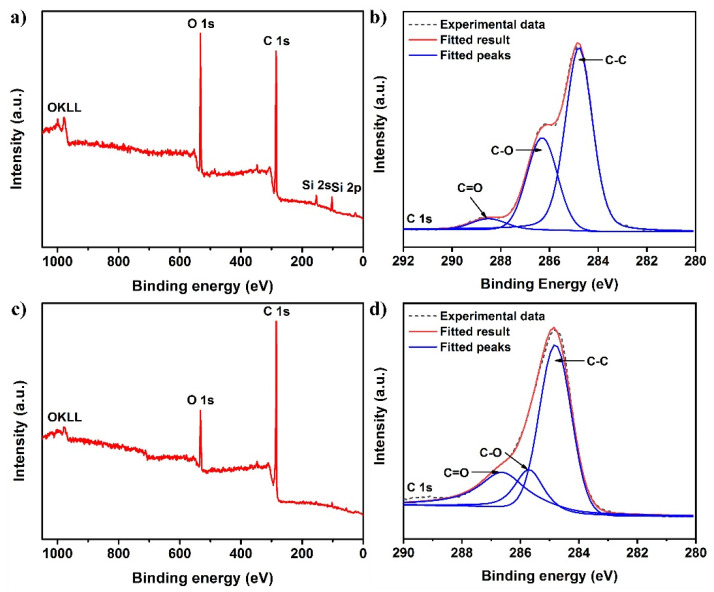
(**a**) XPS survey spectrum and (**b**) high-resolution C 1s spectra of glued SSM. (**c**) XPS survey spectrum and (**d**) high-resolution C 1s spectra of CS-coated mesh.

In addition, the surface chemical composition of the as-fabricated samples was investigated by XPS. The peaks of O 1s, C 1s, Si 2s, and Si 2p were detected in the XPS survey spectrum of glued SSM, confirming that the glue successfully covered the mesh surface (Figure 4a). The high-resolution C 1s spectra (Figure 4b) was decomposed into two main peaks, including C-C (284.8 eV) and C-O (286.3 eV) of silane-modified polyether. In contrast, the notable peaks of Si at 153.4 and 102.4 eV almost all disappeared because of the presence of the CS coating (Figure 4c). Moreover, the strong C 1s peak in the XPS survey spectrum depicted that the CS-coated mesh had a higher content of C than that of the glued SSM. The high-resolution C 1s spectra showed that the C=O group (286.7 eV) appeared on the CS-coated mesh (Figure 4d), implying that the incomplete burned wax existed in the CS coating. Generally, the introduction of these functional groups with low surface energy acted to increase the hydrophobicity of the as-prepared mesh.

### 3.2. Wettability Characterizations and Durability Evaluation

**Figure 5:**
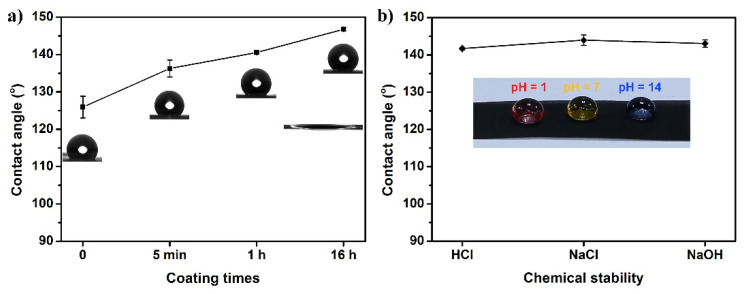
(**a**) Effect of coating times on CAs of the fabricated meshes. The insets show the corresponding CA optical images. (**b**) Influence of acidic, saline, and alkaline environments on the wetting behavior of the CS-coated mesh obtained at 16 h.

Prior to wettability characterization, the samples were fixed on glass slides using double-sided tape. Supplementary Figure S4a shows the semispherical shape of a water droplet on the pristine SSM, revealing that the uncoated SSM exhibits hydrophobicity with a water contact angle (CA) of about 105.2 ± 4.6°. It is reported that the CS-functionalized surface possesses a superhydrophobic characteristic, which is obtained by placing any heat-resistant substrate (stainless-steel, ceramic plate, glass slide, etc.) on top of the mid-flame position of a burning paraffin candle [45]. However, the weak interaction between the CS and the substrate causes the CS layer to be unstable and easily removed, necessitating the addition of a low surface energy polymer acting as a binder for improving the mechanical strength. It can be seen that the water CA of glued SSM was 125.9 ± 2.9° as the glue contains hydrophobic compounds (Figure 5a, left). To explore the effect of CS coating times on wettability, the glued SSMs were immersed in CS dispersion for 5 min, 1 h, and 16 h, respectively. At the 5 min coating time, water droplets on the as-prepared mesh could penetrate into the forming structure (Supplementary Figure S2a) and adhere to the mesh surface, with a CA of 136.3 ± 2.3°. Extending the coating time to 1 h, the water CA increased to 140.5 ± 0.1°, as a result of the gradually growing CS coating, but a few uncoated areas still existed at the same time. When the coating time was prolonged to 16 h, the CS correspondingly rose as a function of time and covered the entire wire surface. The water CA reached 146.8 ± 0.5°, while the CCl_4_ droplet placed on the mesh quickly penetrated and spread within 1 s, illustrating that the as-prepared mesh displayed highly hydrophobic (near-superhydrophobic) and superoleophilic properties. It should be noted that there were no other particles doped in the whole fabrication process for enhancing the roughness, and no fluorinated reagent was utilized for supplying the low surface energy substance. In addition, the SSM without glue pretreatment was also immersed into the CS suspension for 16 h. Supplementary Figure S4b shows that a few agglomerates of CS were loaded on the wire substrate, resulting in discrete and friable properties with a low CA of 123.4 ± 1.4°.

It is imperative for superhydrophobic or highly hydrophobic surfaces to be kept robust in practical oil/water separation applications. To systematically evaluate the durability of CS-coated mesh, the resistance to ultrasonic wear, strong acid/alkaline solutions, and a high salt environment were investigated. When the fabricated mesh was deliberately immersed in deionized water and sonicated for 2 h, the morphology was nearly unchanged on the wire surface compared with that of the freshly obtained mesh (Figure 2d), and the corresponding water CA decreased a little, to 136.3 ± 0.6° (Supplementary Figure S5). Similarly, after dripping drops of HCl solution (pH = 1), NaOH solution (pH = 14), and NaCl solution (saturated) on the mesh surface, these droplets were stable and exhibited elliptical spheres with CAs all above 140° (Figure 5b), indicating excellent resistance to harsh conditions. The great mechanical robustness and chemical stability are attributed to the superglue for firmly bonding intrinsic low surface energy CS nanoparticles onto the SSM to form a compact and durable highly hydrophobic surface.

### 3.3. Oil/Water Separation Performance

**Figure 6:**
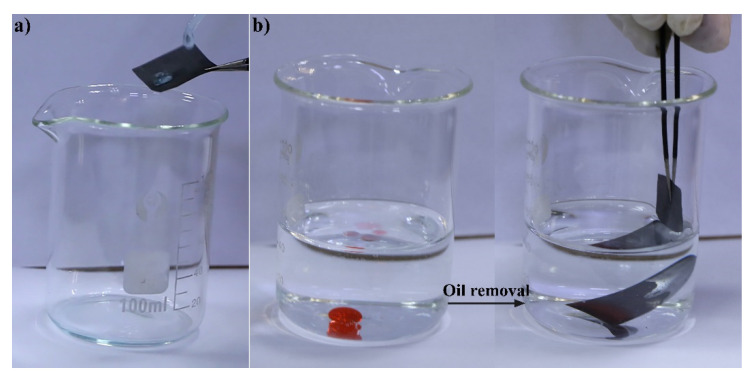
Images of (**a**) continuous water droplets bouncing off the surface of CS-coated mesh, and (**b**) oil removal process of the fabricated mesh towards the CCl_4_-in-water mixture.

As shown in Figure 6a and Supplementary Video S1, continuous water droplets from the pipet readily bounced off the surface of CS-coated mesh, leaving no trace. Moreover, when the mesh was partially immersed under water by an external force, a mirror-like bright surface was observed (Figure 6b), which is in accordance with the Cassie–Baxter wetting behavior mode, where air is trapped between the rough structure of the highly hydrophobic mesh and the surrounding water. The liquid surface that came in contact with the mesh was bent downward, illustrating that the as-fabricated mesh possesses robust hydrophobicity. Once the mesh came in contact with a drop of CCl_4_ dyed with Sudan III, the oil was completely absorbed within a very short time (see Supplementary Video S2). These results confirm that this highly hydrophobic/superoleophilic mesh possesses good oil removal capability. Due to the limited absorption capacity of the mesh, the collection of only a small amount of oil was far from sufficient for practical oily wastewater treatment.

**Figure 7:**
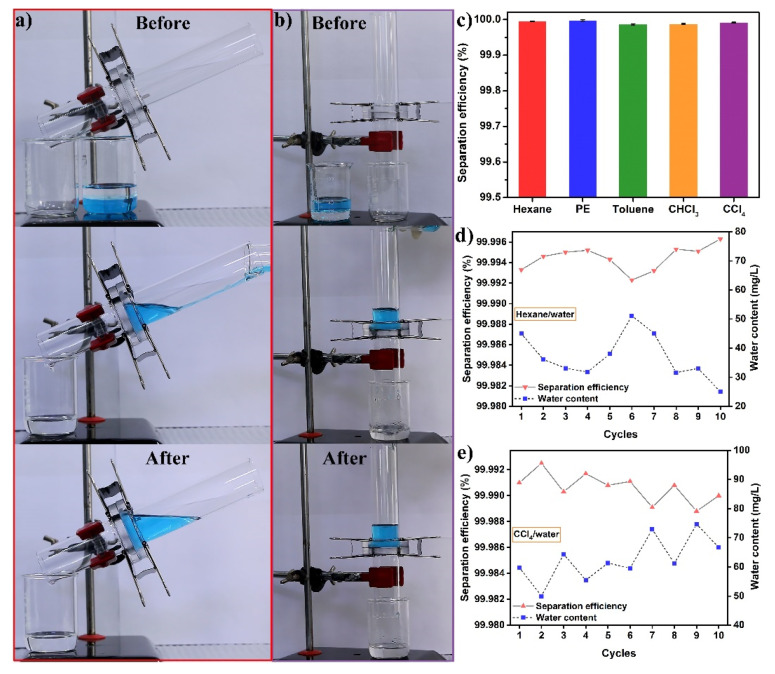
Separation of oil/water mixtures by the CS-coated mesh: The separation processes of (**a**) the light oil (hexane)/water mixture and (**b**) the heavy oil (CCl_4_)/water mixture. The separation efficiency and water contents of (**c**) different oil/water mixtures, and the variation of separation efficiency with (**d**) hexane/water and (**e**) CCl_4_/water separation cycles.

Accordingly, to explore the separation performance of the CS-coated mesh, an experimental setup was built, as shown in Figure 7a,b. Therein, light oils including hexane, PE, and toluene, and heavy oils such as CHCl_3_ and CCl_4_ were used as the simulated oils, while water was dyed with methylene blue. For light oil (hexane)/water separation, the apparatus was inclined by an iron stand support to guarantee that the oil phase encountered the mesh. When the hexane/water mixture was poured onto the mesh which was fixed between two glass tubes, hexane encountered and quickly passed through the mesh, whereas water arrived late but could not penetrate into it due to the prevention of the composite interface composed of the trapped air and the highly hydrophobic surface. Hence, the integration of the porous and rough structure of the SSM with the high hydrophobicity/superoleophilicity enabled the CS-coated mesh to completely separate the light oil/water mixture without any visible hexane floating on the water (Figure 7a and Supplementary Video S3). Considering the existence of huge amounts of viscous oil (e.g., diesel, silicone oil, and crude oil) in real oil spills, dealing with the viscous oily wastewater is an urgent demand [46,47]. Here, we also tested the performance of the CS-coated mesh for separating the diesel/water mixture. As expected, the viscous diesel could be totally purified while water was blocked in the above tube (Supplementary Figure S6). As depicted in Figure 7b, when the high-density CCl_4_/water mixture was dumped into the vertical apparatus, water was effectively repelled and blocked in the upper tube due to the high hydrophobicity of the as-fabricated mesh, while the oil phase could swiftly permeate through the mesh, achieving successful heavy oil/water separation (see Supplementary Video S4). The treatments of other immiscible oil (toluene, PE, or CHCl_3_)/water mixtures were similarly carried out and the separation efficiency was correspondingly calculated. Figure 7c shows that the values for various oil/water mixtures were higher than 99.95%, signifying the efficient separation performance without an external driving force. Another significant feature showing the successful design of CS-coated mesh is the durability. As shown in Figure 7d,e, the reusability of the mesh was characterized by duplicating the separation of the hexane/water mixture and the CCl_4_/water mixture 10 times, respectively. Notably, the stable separation performance was confirmed by a separation efficiency towards the hexane/water mixture of larger than 99.99%, and the separation efficiency of the CCl_4_/water mixture during 10 separation cycles remained above 99.99%. Furthermore, the surface morphology and water CA of the mesh after being repeatedly utilized for 20 cycles were tested. Supplementary Figure S7 shows that there were no changes observed on the mesh surface and the CA remained larger than 143.1 ± 2.7°. All of these characterizations demonstrate that the CS-coated mesh possesses durable oil/water separation performance, which benefits from its structural stability and mechanical robustness.

## 4. Conclusions

In summary, we have presented an inexpensive and facile method to fabricate a highly hydrophobic, onion-like candle soot-coated mesh for versatile oil/water separation, with excellent reusability and durability. Taking advantage of a superglue acting as a binder, the CS coating is close-packed and ultrathin, with a thickness of about 750 nm. The interconnected and intrinsic hydrophobic carbon nanoparticles anchored on the micron wires enabled the mesh to be highly hydrophobic and superoleophilic, as well as to resist harsh environmental conditions, including acid, alkali, and salt solutions, and even ultrasonic wear. The as-prepared mesh was shown to efficiently separate light or heavy oil/water mixtures with a separation efficiency higher than 99.95%, among which the water content in all the oil filtrates was below 75 ppm. Additionally, the mesh maintained an excellent separation performance and high hydrophobicity even after 20 cyclic separation tests, demonstrating its superior reusability and durability. Overall, this work not only showed that the CS-coated mesh is promising for efficient and durable oil/water separation, but also put forward an eco-friendly approach to develop robust superhydrophobic surfaces.

## Data Availability

The data is available on reasonable request from the corresponding author.

## Supplementary Materials

The following supporting information can be downloaded at: https://www.mdpi.com/article/10.3390/nano12050761/s1, Figure S1: (a) SEM image of glued SSM, (b–d) corresponding elemental mapping images of glued SSM. The scale bar is 1 μm. Figure S2: SEM images of the fabricated meshes at different coating times: (a) 5 min and (b) 1 h. Figure S3: Nitrogen adsorption/desorption isotherm curve of CS powders. Figure S4: SEM images of (a) the pristine SSM and (b) CS-coated SSM without glue pretreatment, insets show the corresponding water CAs. Figure S5: SEM image of CS-coated mesh after ultrasonic wear for 2 h, inset shows the corresponding water CA. Figure S6: Separation process of diesel/water mixture by the CS coated mesh. Figure S7: SEM image of CS coated mesh after being repeatedly utilized for 20 cycles of oil/water separation tests, inset was the corresponding water CA. Video S1: Continuous water droplets from the pipet readily bounced off the surface of CS-coated mesh. Video S2: Removal process of a drop of CCl_4_ dyed with Sudan III from water. Video S3: Separation process of the light oil (hexane)/water mixture. Video S4: Separation process of the heavy oil (CCl_4_)/water mixture.
