# Supplementary material for: Facile Fabrication of Highly Hydrophobic Onion-like Candle Soot-Coated Mesh for Durable Oil/Water Separation
Source: Nanomaterials (Basel). 2022 Feb 24;12(5):761. doi: 10.3390/nano12050761 (PMC8912305; doi:10.3390/nano12050761)
Supplement: Supplementary file 1 [file nanomaterials-12-00761-s001.zip › nanomaterials-1568079-Supplementary/nanomaterials-1568079-Supplementary.pdf]

Supplementary Materials

Facile Fabrication of Highly Hydrophobic Onion-Like Candle Soot Coated Mesh for Durable Oil/Water Separation

Jiajia Song [1,†], Na Liu [2,†], Jiakai Li [1], Yingze Cao [3,*] and Haijie Cao [1,*]

1. Institute of Materials for Energy and Environment, College of Materials Science and Engineering, Qingdao University, Qingdao 266071, China; songjiajia540819@163.com (J.S.); 2019025733@qdu.edu.cn (J.L.)
2. Department of Chemistry, Tsinghua University, Beijing 100084, China; liun12@tsinghua.org.cn (N.L.);
3. Qian Xuesen Laboratory of Space Technology, China Academy of Space Technology, Beijing 100094, China

* Correspondence: caoyingze@qxslab.cn (Y.C.); caohj1582@hotmail.com (H.C.)

† These authors contributed equally to this work.

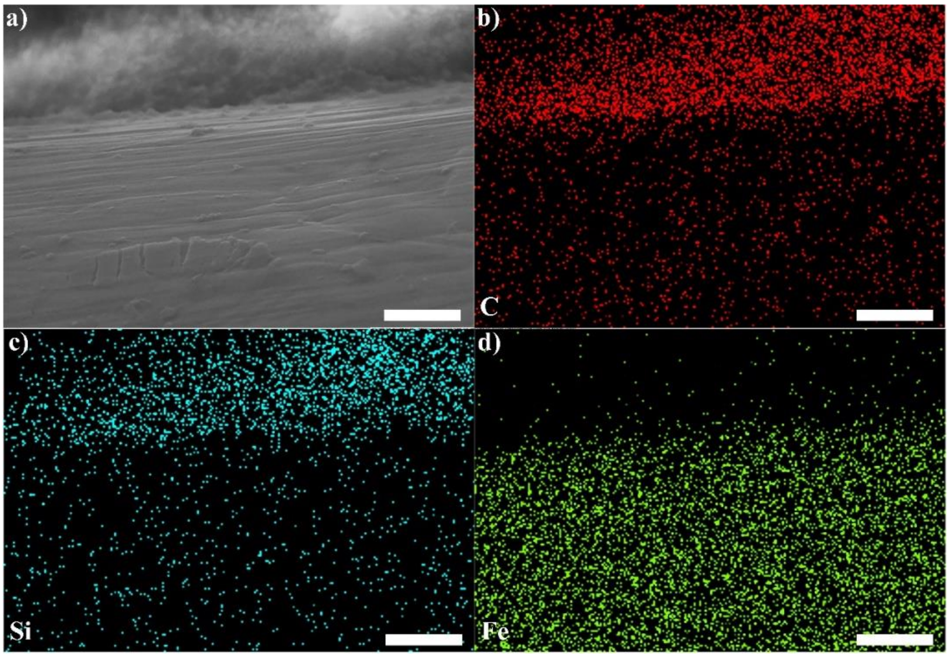

Figure S1. (a) SEM image of glued SSM. (b-d) Corresponding elemental mapping images of glued SSM. The scale bar is 1 μm.

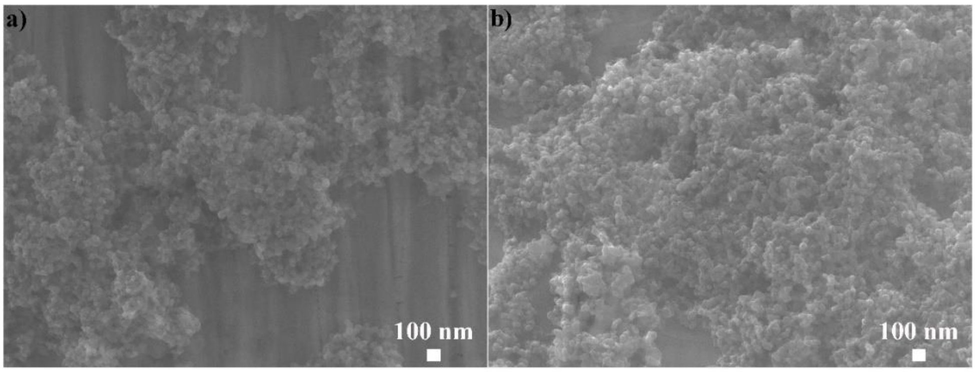

Figure S2. SEM images of the fabricated meshes at different coating times: (a) 5 min and (b) 1 h.

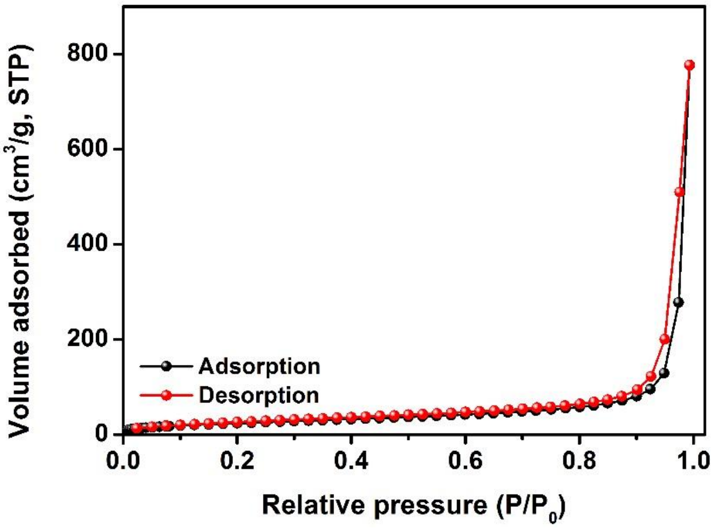

Figure S3. Nitrogen adsorption/desorption isotherm curve of CS powders.

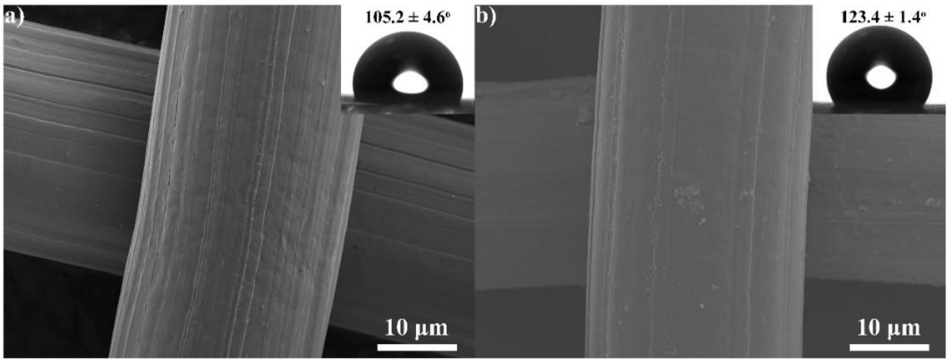

Figure S4. SEM images of (a) the pristine SSM and (b) CS coated SSM without glue pretreated, insets were the corresponding water CAs.

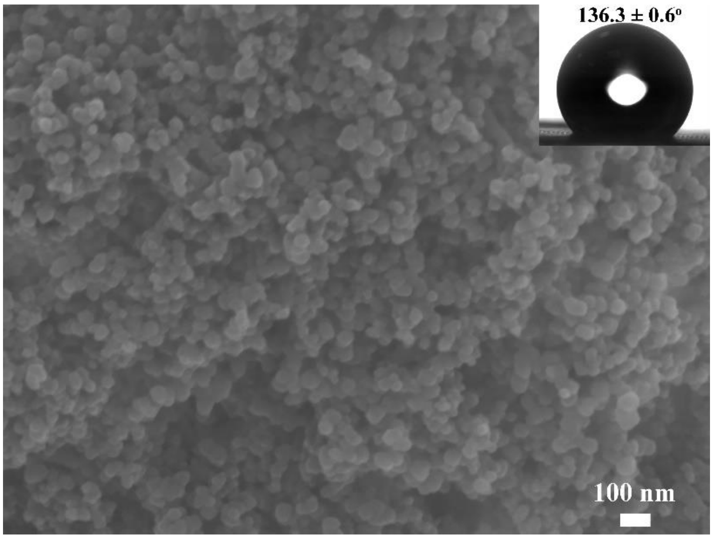

Figure S5. SEM image of CS coated mesh after ultrasonic worn for 2 h, inset was the corresponding water CA.

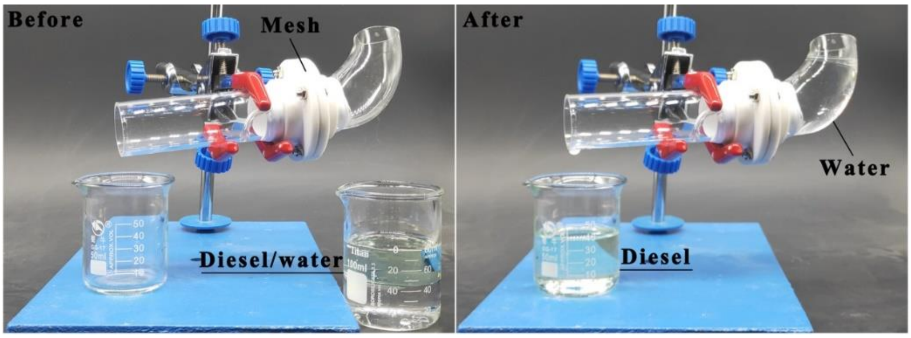

Figure S6. Separation process of diesel/water mixture by the CS coated mesh.

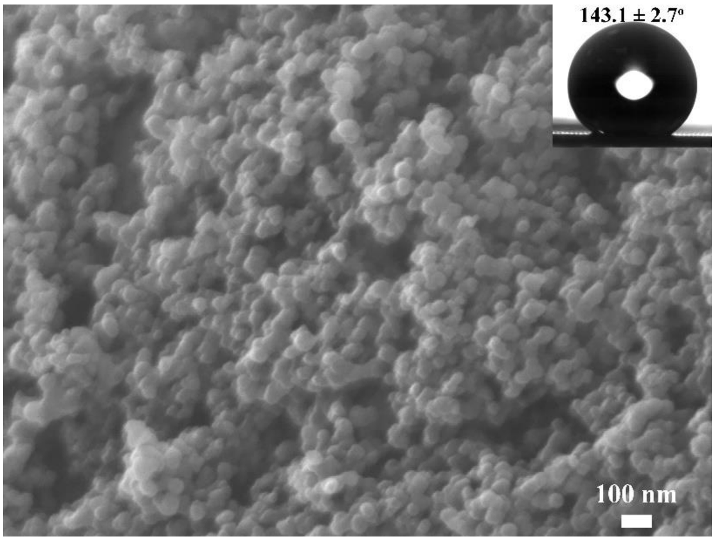

Figure S7. SEM image of CS coated mesh after being repeatedly utilized for 20 cycles of oil/water separation tests, inset was the corresponding water CA.

Video S1. Continuous water droplets from the pipet readily bounced off the surface of CS coated mesh.

Video S2. Removal process of a drop of CCl_4 dyed with Sudan III from water.

Video S3. Separation process of light oil (hexane)/water mixture.

Video S4. Separation process of heavy oil (CCl_4)/water mixture.
